# Efficacy and safety of cinobufacini capsules combined with oxaliplatin-based chemotherapy for advanced colorectal cancer: a human systematic review and meta-analysis

**DOI:** 10.3389/fphar.2026.1709044

**Published:** 2026-01-30

**Authors:** Jian Wang, Qijia Gao, Yangming Que, Dingting Zheng, Jianxin Chen

**Affiliations:** 1 M.D. Department of Gastroenterology, Jiaxing Second Hospital, Jiaxing, Zhejiang, China; 2 Changsha Medical University, Changsha, Hunan, China; 3 M.D. Department of Gastroenterology, The Second People’s Hospital of Quzhou, Quzhou, Zhejiang, China; 4 M.D. Department of Education, International Word, Quzhou People′s Hospital, The Quzhou Affiliated Hospital of Wenzhou Medical University, Quzhou, Zhejiang, China

**Keywords:** chemotherapy, cinobufacini capsules, colorectal cancer, meta-analysis, traditional Chinese medicine

## Abstract

**Background:**

Patients with stage III–IV colorectal cancer (CRC) face poor prognosis due to metastases and limited treatment options. In China, cinobufacini capsules are widely used as a complementary medicine, but evidence for their clinical value remains insufficient.

**Methods:**

Systematic searches were conducted across seven databases, including Chinese National Knowledge Infrastructure (CNKI), WanFang Database, China Biological Medicine Database (CBM), PubMed, Embase, the Cochrane Library, and Web of Science, from their inception to 30 June 2025. The primary outcome was disease control rate (DCR), and the secondary outcomes were objective response rate (ORR), CD4^+^ T cells, carcinoembryonic antigen (CEA), carbohydrate antigen 19-9 (CA19-9), carbohydrate antigen 125 (CA-125), and adverse reactions. This study strictly followed the PRISMA guidelines and used RevMan 5.3, Stata 15, and GRADEpro for data analysis.

**Results:**

The analysis showed that the combination therapy resulted in a higher DCR (RR = 1.18, 95% CI: 1.10–1.25; *P* < 0.0001) and ORR (RR = 1.40, 95% CI: 1.23–1.60; *P* < 0.0001), reduced CEA levels (WMD = −7.84, 95% CI: −10.43 to −5.23; *P* < 0.0001), CA-125 levels (WMD = −9.36, 95% CI: −12.80 to −5.92; *P* < 0.0001), and CA19-9 levels (WMD = −11.38, 95% CI: −12.66 to −10.11; *P* < 0.0001), as well as higher CD4^+^ T-cell levels (WMD = 2.74, 95% CI: 1.99–3.49; *P* < 0.0001). Additionally, the combination therapy could decrease the incidence of adverse reactions including leukocyte toxicity (RR = 0.59, 95% CI: 0.48–0.71; *P* < 0.0001), gastrointestinal toxicity (RR = 0.79, 95% CI: 0.62–0.99; *P* = 0.03), and myelosuppression (RR = 0.65, 95% CI: 0.46–0.92; *P* = 0.01). The level of evidence was assessed as moderate for DCR and low for ORR.

**Conclusion:**

Cinobufacini combined with oxaliplatin-based chemotherapy may enhance short-term efficacy, immune function, and safety in advanced CRC. However, due to limitations of existing RCTs, these findings should be interpreted cautiously, and further large-scale, high-quality trials are required.

## 1 Introduction

Colorectal cancer (CRC) is the third most common malignancy worldwide and the second leading cause of cancer-related death, following lung cancer, accounting for approximately 9.4% of cancer mortality (Alese et al., 2023; Strickler et al., 2022). In 2023, there were approximately 153,020 newly diagnosed CRC patients in America alone, with an estimated death toll of 52,550 (Siegel et al., 2023). Among new cases of colorectal cancer, approximately 20% present with metastasis, and more than 60% of those under 50 years old are diagnosed at an advanced stage (Tumor–Node–Metastasis [TNM] stages III–IV) (Biller and Schrag, 2021; Fass et al., 2020). Furthermore, the prognosis of CRC varies significantly across different tumor stages (Hernandez Dominguez et al., 2023). Stage I patients have a 90% 5-year survival rate, while stage IV patients have a significantly lower rate of only 15.1% due to the limited treatment options and the burden of therapy-resistant, metastasis-competent cancer cells (Hernandez Dominguez et al., 2023; Weng and Goel, 2022; Shin et al., 2023). Given the high mortality and the trend of younger individuals being affected in CRC, early detection and intervention are increasingly recognized as critical components in the management of CRC (Patel et al., 2022). Owing to limited screening awareness and the insidious nature of CRC, many individuals are already at advanced stages when symptoms such as chronic abdominal pain, hematochezia, and anemia appear, thereby missing the opportunity for surgical intervention. For the majority of patients with advanced colorectal cancer, first-line treatment typically involves oxaliplatin-based chemotherapy regimens, including capecitabine plus oxaliplatin (XELOX), leucovorin (folinic acid), 5-fluorouracil, and oxaliplatin (FOLFOX), leucovorin (folinic acid), 5-fluorouracil, and irinotecan (FOLFIRI), or leucovorin (folinic acid), 5-fluorouracil, oxaliplatin, and irinotecan (FOLFOXIRI) (Innocenti et al., 2021). These regimens could be used as monotherapy or in conjunction with targeted therapy or immunotherapy drugs tailored to the patient’s molecular subtypes, aiming to extend overall survival (Biller and Schrag, 2021; Feria and Times, 2024). However, oxaliplatin-based chemotherapy, a cornerstone in the treatment of colorectal cancer, is commonly associated with hematological toxicities, gastrointestinal adverse events, and cumulative peripheral neurotoxicity (Biller and Schrag, 2021; Kokotis et al., 2016). These adverse reactions weaken the patient’s immune function, degrade their quality of life, and increase the risk of interrupting the continuity of treatment (Zraik and Heß-Busch, 2021). Complicating matters further, many advanced CRC patients also grapple with cachexia—a condition characterized by muscle wasting, weakness, and fatigue. This prevalent condition, observed in 50%–61% of CRC cases, independently reduces overall survival (OS) (Kasprzak, 2021). It is crucial to note that chemotherapy could exacerbate the symptoms of cachexia, creating a vicious cycle that further impacts the prognosis of patients (Ferrer et al., 2023). Therefore, there is a recognized need for additional treatments to prolong the survival outcomes and decrease the toxicity of chemotherapy for patients with advanced CRC.

Cinobufacini, also known as Huachansu in Chinese, is a single-herb traditional Chinese medicine preparation extracted from the dried skin of the toad *Bufo gargarizans* Cantor (family Bufonidae). It is formulated as an oral capsule, with bufadienolides (such as bufalin, cinobufagin, and resibufogenin) identified as the principal bioactive antitumor components (Zhan X. et al., 2020; Li R. et al., 2022; Wei et al., 2017). A randomized controlled study revealed that incorporating cinobufacini capsules (CC) into the first-line treatment for postoperative patients with stage II-III CRC could extend the average 3-year disease-free survival (DFS) by approximately 4 months (L et al., 2023). This treatment approach also resulted in a decrease in adverse reactions, including leukopenia, neutropenia, and diarrhea. Furthermore, supportive evidence from a recent breast cancer meta-analysis suggests that CC may have the effect of prolonging life and improving chemotherapy tolerance (Wu et al., 2025). Currently, cinobufacini capsules have been approved by the China National Medical Products Administration (NMPA) as complementary agents in various cancers, including but not limited to liver cancer, non-small cell lung cancer, and colorectal cancer; however, they are not approved by the U.S. Food and Drug Administration or the European Medicines Agency and are regarded as complementary or alternative medicine. In addition, with the growing research interest in cinobufacini in recent years, it is necessary to systematically evaluate its overall efficacy in colorectal cancer. Thus, this meta-analysis seeks to provide a comprehensive evaluation of cinobufacini capsules in treating advanced CRC, offering a new supplementary approach for patients in need.

## 2 Materials and methods

The study followed the Preferred Reporting Items for Systematic Review and Meta-analysis (PRISMA) guidelines (Page et al., 2021) and was registered in PROSPERO (CRD42024529353). The registered outcomes were retained, with the only protocol modification being an extension of the literature search end date to 30 June 2025. Ethical approval was not required as this study used published data only.

### 2.1 Database searching

A comprehensive search strategy was employed to retrieve relevant studies from seven literature databases, including Chinese National Knowledge Infrastructure (CNKI), WanFang Database, China Biological Medicine Database (CBM), PubMed, Embase, The Cochrane Library, and Web of Science (WOS). The search period ranged from the inception of each database to 30 June 2025. The search terms listed in Table 1 were used for English databases, while the corresponding Chinese terminology was utilized when searching Chinese databases.

**TABLE 1:** The search strategies of the English database.

#1	“Colorectal Neoplasms”[Mesh]
#2	(((((((((((((((“Colorectal Neoplasms”[Mesh]) OR (Colorectal Neoplasm[Title/Abstract])) OR (Neoplasm, Colorectal[Title/Abstract])) OR (Neoplasms, Colorectal[Title/Abstract])) OR (Colorectal Tumors[Title/Abstract])) OR (Colorectal Tumor[Title/Abstract])) OR (Tumor, Colorectal[Title/Abstract])) OR (Tumors, Colorectal[Title/Abstract])) OR (Colorectal Cancer[Title/Abstract])) OR (Cancer, Colorectal[Title/Abstract])) OR (Cancers, Colorectal[Title/Abstract])) OR (Colorectal Cancers[Title/Abstract])) OR (Colorectal Carcinoma[Title/Abstract])) OR (Carcinoma, Colorectal[Title/Abstract])) OR (Carcinomas, Colorectal[Title/Abstract])) OR (Colorectal Carcinomas[Title/Abstract])
#3	(Cinobufacini[Title/Abstract]) OR (HuaChanSu[Title/Abstract])
#4	#3 AND #2

### 2.2 Inclusion criteria

Studies were included if they met the following criteria: randomized controlled trials (RCTs) design; patients with histologically or cytologically confirmed stage III or IV colorectal cancer; comparison between oxaliplatin-based chemotherapy alone and oxaliplatin-based chemotherapy combined with oral cinobufacini capsules; and comparable baseline characteristics between treatment groups. Eligible studies were required to report at least one relevant clinical outcome, including disease control rate (DCR) or objective response rate (ORR), as well as immunological indices, tumor markers, or treatment-related adverse events.

### 2.3 Exclusion criteria

Studies were excluded if they were duplicate publications, had incomplete or unclear data, or insufficiently described interventions. Non-randomized studies were excluded, as were studies involving patients without clearly defined stage III or IV colorectal cancer. Trials in which cinobufacini was administered via non-oral routes were also excluded.

### 2.4 Quality assessment and data extraction

Two qualified researchers independently extracted relevant data from studies that met the inclusion criteria, including authors, year of publication, sample size, tumor response criteria, interventions, duration, and outcomes. The risk of bias in these studies was assessed using the Cochrane collaboration tool, including evaluations of the study’s randomization process, implementation of blinding, integrity of trial data, selective reporting of results, and other biases. The studies were categorized as low, high, or unclear risk of bias. Any disagreements regarding data extraction and assessment of bias risk were resolved by the corresponding author.

### 2.5 Data analysis

The data extracted from the studies were analyzed using RevMan 5.3 and Stata 15 software. For dichotomous data, Risk Ratios (RR) were employed as measures of effect size, while continuous data were represented by weighted mean difference (WMD) or standardized mean difference (SMD). Each effect size was expressed as a 95% confidence interval (CI). A fixed-effects analysis model was utilized when heterogeneity was at or below 50% (I^2^ ≤ 50%), and a random-effects model was adopted for I^2^ > 50%. If there was significant heterogeneity, sensitivity analysis was used to assess the stability of the results. If the studies included for the outcome indicator were ≥10, publication bias was assessed using funnel plots and Begg’s/Egger’s test. Meta-regression analysis was employed to assess the influence of various factors on the outcomes. The quality of evidence was assessed using GRADE software.

## 3 Results

### 3.1 Search results and study characteristics

**FIGURE 1:**
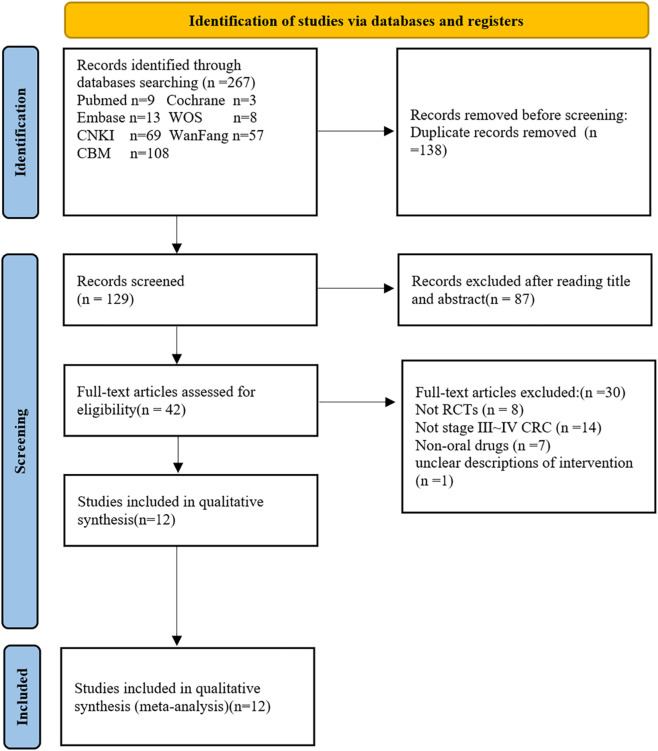
Flow diagram of the study.

As depicted in Figure 1, the literature search spanned several databases, including WOS, CNKI, WanFang, PubMed, Cochrane, Embase, and CBM, resulting in a total of 267 records. After removing 138 duplicate entries, screening of titles and abstracts excluded 87 articles, leaving 42 for full-text review. Among these, thirty studies were further excluded for reasons including not being RCTs, not involving stage III-IV colorectal cancer, using non-oral drugs, or having unclear intervention descriptions. Finally, twelve studies (Yang and Peng, 2021; Zhong et al., 2019; Song, 2022; Cheng et al., 2023; Dong et al., 2017; Fang G. et al., 2021; Zhang, 2021; Wang, 2023; Shi et al., 2017; Lu et al., 2014; Zhou, 2023; Sha et al., 2018) with 954 cases were ultimately included in this meta-analysis. The general characteristics of the included studies are detailed in Table 2.

**TABLE 2:** Principal characteristics of RCTs included in the meta-analysis.

study (year)	Age (years)	C	Sample size	Tumor responses criteria	Stage	Intervention		Cinobufacini dose	Duration (cycles)	Outcome measures
	T		(T/C)			T	C			
Zhou, 2023	57.89 ± 6.12	58.47 ± 6.25	43/43	RECIST	III,IV	XELOX + CC	XELOX	0.5g-tid-po	2	①②⑤
Cheng, 2023	53.42 ± 7.34	52.63 ± 7.21	31/31	NA	IIIB,IV	FOLFOXIRI + CC	FOLFOXIRI	0.5g-tid-po	6	③④
Song, 2022	61.98 ± 5.77	62.15 ± 5.87	50/50	RECIST	III,IV	FOLFOX + CC	FOLFOX	0.5g-bid-po	4	①②③④⑤
Zhang, 2021	53.36 ± 7.43	53.41 ± 7.46	36/36	RECIST	III,IV	XELOX + CC	XELOX	0.5g-tid-po	2	①②⑤
Yang, 2021	58.30 ± 7.89	59.61 ± 7.93	31/31	RECIST	III,IV	XELOX + CC	XELOX	0.5g-tid-po	6	①②③⑤
Zhong, 2019	62-82	64-78	44/42	RECIST	III,IV	XELOX + CC	XELOX	0.75g-tid-po	2	①②⑤
Sha, 2018	42-70	43-72	41/41	NA	III,IV	PTX + OX + CC	PTX + OX	0.5g-tid-po	2	③④
Dong, 2017	58. 6 ± 7. 4	60.2 ± 6.8	38/38	Unclear	III,IV	XELOX + CC	XELOX	0.5g-tid-po	4	①②⑤
Shi, 2017	35. 57 ± 2. 35	35.42 ± 4. 8	34/34	RECIST	III	FOLFOX + CC	FOLFOX	0.6g-bid-po	4	①②⑤
Lu, 2014	38–75	36–73	30/30	RECIST	III,IV	FOLFOX + CC	FOLFOX	0.5g-tid-po	2	①②⑤
Wang, 2023	58.14 ± 7.31	57.89 ± 7.03	40/40	WHO	IIIB,IV	XELOX + CC	XELOX	0.5g-tid-po	4	①②③④
Fang, 2021	68.72 ± 3.21	68.80 ± 3.19	60/60	RECIST	IIIB,IV	XELOX + CC	XELOX	0.5g-tid-po	4	①②③

C: control group, T: treatment group, NA: not available, RECIST: response evaluation criteria in solid tumors, WHO:World Health Organization XELOX: Xeloda + Oxaliplatin, FOLFOXIRI: Leucovorin+5-Fluorouracil + Oxaliplatin + Irinotecan, FOLFOX: Leucovorin+5-Fluorouracil + Oxaliplatin, PTX + OX: Raltitrexed + Oxaliplatin, CC: cinobufacini capsule, Outcome measures: ①: DCR, ②: ORR, ③: Tumor Markers, ④: CD4 T cells, ⑤: Adverse Events.

### 3.2 Risk of bias

**FIGURE 2:**
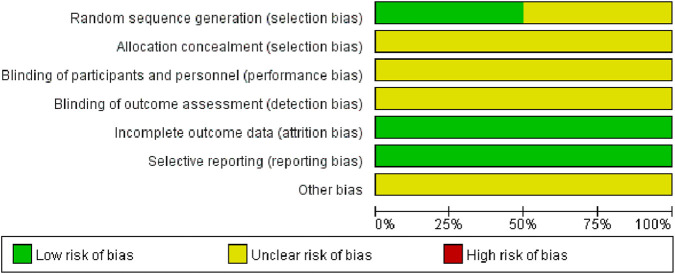
Risk of bias graph.

**FIGURE 3:**
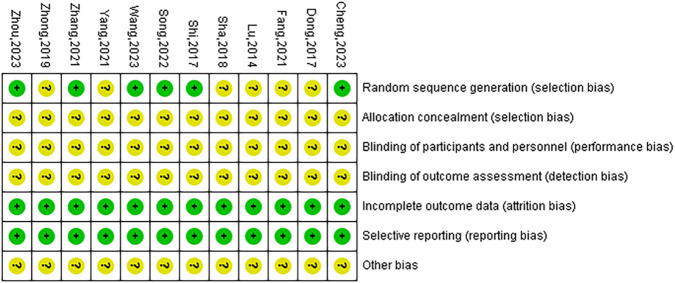
Risk of bias summary.

While all included articles mentioned the use of randomization methods, only 6 (Cheng et al., 2023; Dong et al., 2017; Zhang, 2021; WANG, 2023; Shi et al., 2017; Zhou, 2023) articles provided detailed descriptions of their randomization procedures. The blinding of participants, personnel, and outcome assessment in all included RCTs was unclear. However, all studies provided complete data, and no selective bias was discovered. Additionally, it was not possible to ascertain the presence of any other biases, which were therefore assessed as ‘unclear’ (Figures 2, 3).

### 3.3 Outcome measures

#### 3.3.1 Disease control rate and objective response rate

**FIGURE 4:**
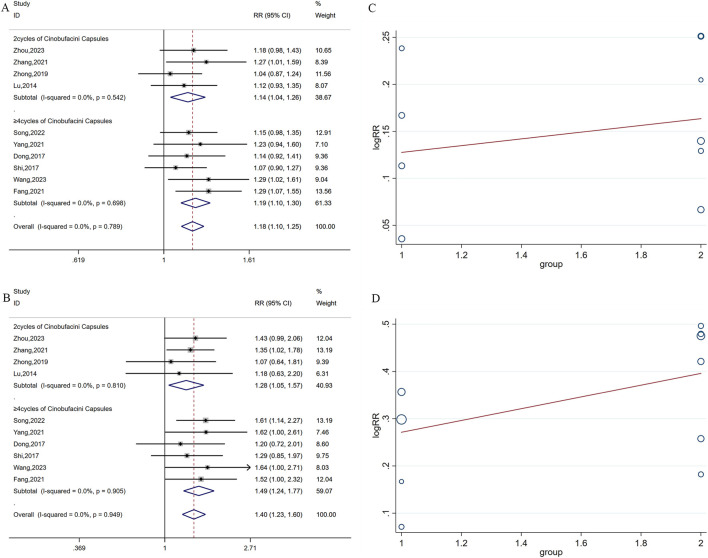
Forest plots of DCR **(A)** and ORR **(B)**; Meta-regression analysis plots of DCR **(C)** and ORR **(D)**.

Ten studies respectively reported DCR and ORR, including 810 cases. Statistical homogeneity was observed for both outcomes (I^2^ = 0%, *P* = 0.789, *P* = 0.949, respectively), and the fixed-effects model was applied. The combination of cinobufacini capsules and oxaliplatin-based chemotherapy had a remarkable improvement in DCR (RR = 1.18, 95% CI: 1.10–1.25, *P* < 0.0001; Figure 4A) and ORR (RR = 1.40, 95% CI: 1.23–1.60, *P* < 0.0001; Figure 4B) compared to oxaliplatin-based chemotherapy alone.

Because only one study reported a treatment duration of 6 cycles, all studies were divided into 2 cycles (group 1) and ≥4 cycles (group 2) based on the treatment cycles. Subgroup analysis was performed to assess the impact of different treatment cycles of CC on DCR and ORR. Based on the findings, 2 cycles and ≥4 cycles of the combined treatment both enhanced the DCR (RR = 1.14, 95% CI: 1.04–1.26, *P* < 0.0001; RR = 1.19, 95% CI: 1.10, 1.30, *P* < 0.0001, respectively) and ORR (RR = 1.28, 95% CI: 1.05–1.57, *P* < 0.0001; RR = 1.49, 95% CI: 1.24–1.77, *P* < 0.0001, respectively). Additionally, meta-regression analysis showed that the treatment cycle did not affect DCR (*P* = 0.590) or ORR (*P* = 0.379). However, the results depicted in Figures 4C,D indicated a slight increase in tendency with an increased cycle number of cinobufacini capsules.

#### 3.3.2 Tumor markers

**FIGURE 5:**
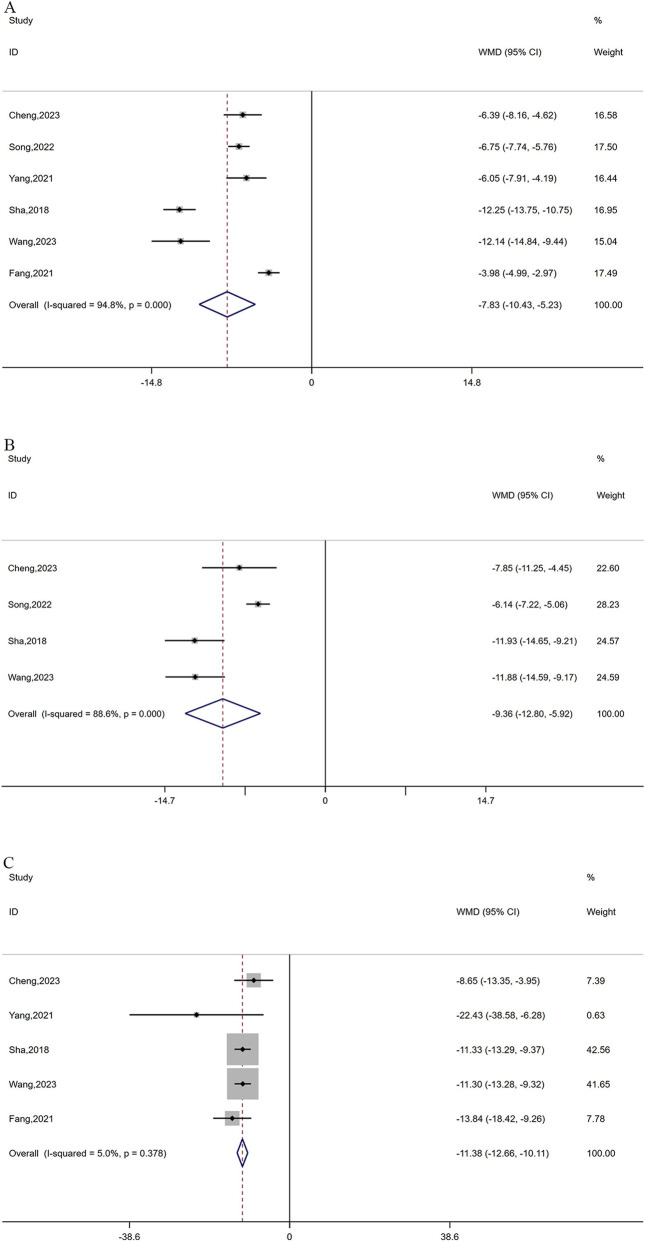
Forest plots of CEA **(A)**, CA-125 **(B)**, and CA19-9 **(C)**.

A total of 6 studies reported CEA levels (Yang and Peng, 2021; Song, 2022; Cheng et al., 2023; Fang K. et al., 2021; Wang, 2023; Sha et al., 2018) and 4 studies (Song, 2022; Cheng et al., 2023; Wang, 2023; Sha et al., 2018) reported CA-125 levels between the treatment group and control group, with 506 and 324 patients, respectively. Because of the significant heterogeneity in CEA and CA-125 (I^2^ = 94.8% for CEA and 88.4% for CA-125, *P* ≤ 0.0001), a random-effects model was utilized. As depicted in Figures 5A,B, oxaliplatin-based chemotherapy combined with cinobufacin capsules can effectively reduce CEA (WMD = −7.84, 95% CI: 10.43 to −5.23, *P* < 0.0001) and CA-125 levels (WMD = −9.36, 95% CI: 12.80 to −5.92, *P* < 0.0001). Additionally, five studies (Yang and Peng, 2021; Cheng et al., 2023; Fang G. et al., 2021; Wang, 2023; Sha et al., 2018) with 406 patients reported CA19-9 levels, suggesting that combination therapy can lead to a reduction in CA19-9 levels (WMD = −11.38, 95% CI: 12.66 to −10.11, *P* < 0.0001; Figure 5C) with no observed heterogeneity (I^2^ = 5%, *P* = 0.378).

#### 3.3.3 CD4 T cells

**FIGURE 6:**
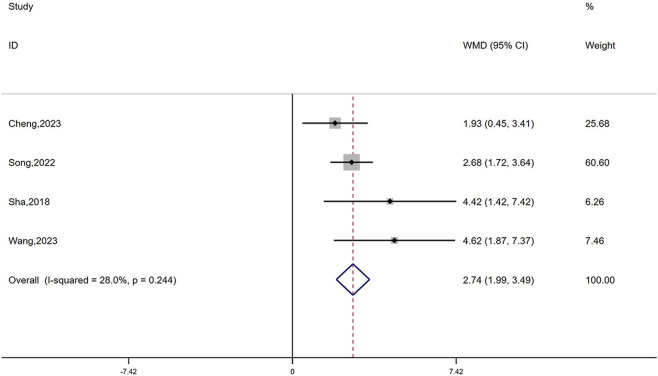
Forest plot of CD4 T cells.

The expression levels of CD4 T cells (Song, 2022; Cheng et al., 2023; Wang, 2023; Sha et al., 2018) were reported in four studies, including 324 cases. A fixed-effects model was adopted based on low heterogeneity (I^2^ = 28%, *P* = 0.244). The results suggested that the combination therapy has a remarkable positive effect on increasing CD4 levels (WMD = 2.74, 95% CI: 1.99–3.49, *P* < 0.0001; Figure 6).

#### 3.3.4 Adverse events

**FIGURE 7:**
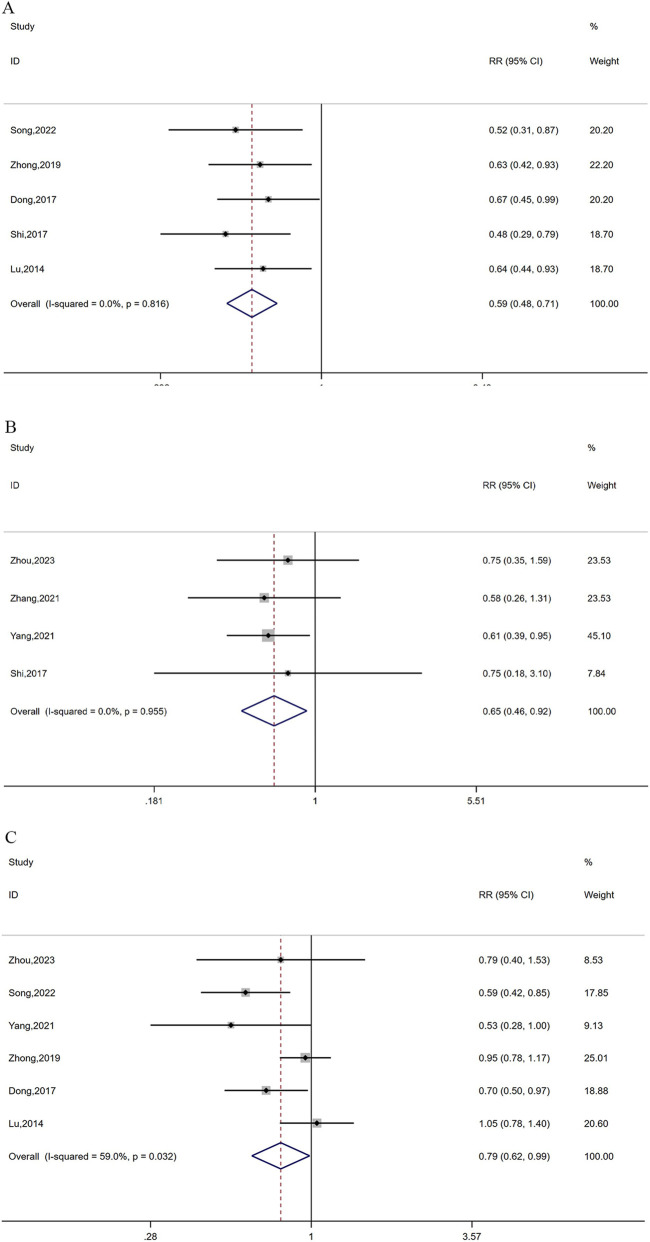
Forest plots of leukocyte toxicity **(A)**, Myelosuppression **(B)**, and gastrointestinal toxicity **(C)**.

Five studies (Zhong et al., 2019; Song, 2022; Dong et al., 2017; Shi et al., 2017; Lu et al., 2014) with 390 patients reported leukocyte toxicity. As no heterogeneity was observed in this study (I^2^ = 0%, *P* = 0.820), a fixed-effects model was applied. The analysis revealed that combination therapy can effectively reduce leukocyte toxicity in patients with advanced CRC (RR = 0.59, 95% CI: 0.48, 0.71, *P* < 0.0001; Figure 7A).

Myelosuppression was described in four studies (Yang and Peng, 2021; Zhang, 2021; Shi et al., 2017; Zhou, 2023) comprising 288 cases. No heterogeneity was observed (I^*2*^ = 0%, *P* = 0.955), and a fixed-effects model was finally applied. The analysis showed that compared with Oxaliplatin-based chemotherapy alone, the combination therapy markedly decreased the occurrence of myelosuppression (RR = 0.65, 95% CI: 0.46–0.92, *P* = 0.01; Figure 7B).

Adverse effects of gastrointestinal toxicity were reported in 6 references (Yang and Peng, 2021; Zhong et al., 2019; Song, 2022; Zhang, 2021; Lu et al., 2014; Zhou, 2023) including 470 cases. Owing to substantial heterogeneity (I^2^ = 59%, *P* = 0.032), a random-effects model was employed. It was found that cinobufacini capsules plus oxaliplatin-based chemotherapy drastically reduced the number of patients with gastrointestinal toxicity when compared with oxaliplatin-based chemotherapy alone. (RR = 0.79, 95% CI: 0.62–0.99, *P* = 0.04; Figure 7C).

#### 3.3.5 Sensitivity analysis

**FIGURE 8:**
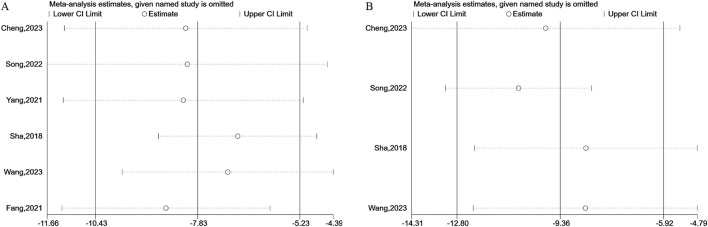
Sensitivity analysis plots of CEA **(A)** and CA-125 **(B)**.

In this study, significant heterogeneity was observed in the levels of CEA and CA-125. After conducting sensitivity analyses, no source of heterogeneity was found, which indicated the reliability of the results (Figures 8A,B).

#### 3.3.6 Publication bias

**FIGURE 9:**
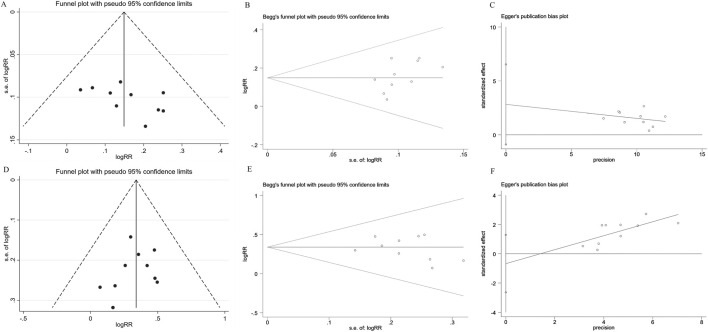
Publication bias plots. Funnel plot **(A)**; Begg plot **(B)**; Egger plot **(C)** of DCR; Funnel plot **(D)**; Begg plot **(E)**; Egger plot **(F)** of ORR.

Publication bias of DCR and ORR was assessed through funnel plots and Begg’s/Egger’s tests. The funnel plot for DCR exhibited slight asymmetry, but both Begg’s test (z = 1.52, *P* = 0.152) and Egger’s test (t = 1.75, *P* = 0.118) indicated the absence of publication bias (Figures 9A–C). However, a potential publication bias was suggested by a minor imbalance on the ORR funnel plot. Begg’s test (z = - 1.16, *P* = 0.283) and Egger’s test (t = −1.80, *P* = 0.450) found no evidence of publication bias (Figures 9D–F).

#### 3.3.7 Quality of evidence

As illustrated in Table 3, when comparing the combination of cinobufacini capsules with oxaliplatin-based chemotherapy to oxaliplatin-based chemotherapy alone, the quality of evidence was assessed as moderate for DCR and low for ORR. The GRADE evaluation did not include other secondary outcomes due to the limited number of studies available.

**TABLE 3:** GRADE evidence profile.

Quality assessment							No of patients		Effect		Quality	Importance
Outcomes	Design	Risk of bias	Inconsistency	Indirectness	Imprecision	Other considerations	Trials	Control	Relative (95% CI)	Absolute
DCR (10)	Randomised trials	serious^a^	no serious Inconsistency	no serious Indirectness	no serious Imprecision	none	365/406 (89.9%)	309/404 (76.5%)	RR 1.18 (1.1–1.25)	138 more per 1,000 (from 76 more to 191 more)	⊕⊕⊕Ο MODERATE	CRITICAL
							​	76.5%		138 more per 1,000 (from 77 more to 191 more)		
ORR (10)	Randomised trials	serious^a^	no serious Inconsistency	no serious Indirectness	serious^b^	none	245/406 (60.3%)	174/404 (43.1%)	RR 1.4 (1.23–1.6)	172 more per 1,000 (from 99 more to 258 more)	⊕⊕ΟΟ LOW	CRITICAL
							​	40.7%		163 more per 1,000 (from 94 more to 244 more)		

^a^ All the included RCTs, exhibited certain deficiencies in randomization and blinding.

^b^ The Relative Risk (RR) is significantly higher or lower (RR < 0.75 or RR > 1.25).

## 4 Discussion

The role of complementary therapies in improving treatment tolerance and clinical outcomes remains an important topic in the management of advanced colorectal cancer. In this context, the present meta-analysis examined the available randomized evidence regarding the addition of cinobufacini capsules to oxaliplatin-based chemotherapy. The findings indicate that combining cinobufacini with standard chemotherapy is associated with improved short-term response and a lower incidence of treatment-related adverse events, which may be particularly relevant for patients with stage III–IV disease who often experience cumulative toxicity.

Based on previous network meta-analyses (Tang et al., 2021; Liu et al., 2022), the combination of oral or intravenous cinobufacini with oxaliplatin-based chemotherapy for CRC treatment has shown higher clinical efficacy, a significant reduction in adverse events, and improved immune function. However, the above studies did not focus on the effect of cinobufacini on advanced colorectal cancer. Our study incorporated the latest randomized controlled trials of cinobufacini in patients with stage III–IV CRC, suggesting that cinobufacini may reduce treatment-related toxicity while improving therapeutic efficacy.

Subgroup analysis of ORR and DCR indicated that patients receiving ≥4 cycles of the combined therapy appeared to have higher efficacy. However, this benefit was not statistically supported by exploratory meta-regression analyses, indicating that extended treatment cycles may not significantly enhance efficacy in advanced colorectal cancer. These discrepancies may be attributable to differences in baseline characteristics across studies, as well as the limited number of included studies and relatively small sample sizes. Additionally, sensitivity analyses did not identify any single study as the primary source of heterogeneity in CEA and CA-125 levels, suggesting that the variability likely reflects multifactorial clinical and methodological differences across studies. Moreover, the overall strength of evidence for DCR and ORR was rated as moderate and low, respectively, according to the GRADE approach. Our analysis also revealed that CC significantly reduces the incidence of adverse events, including leukocyte toxicity, myelosuppression, and gastrointestinal toxicity. Additionally, a phase 1 trial (Meng et al., 2009) indicated that cinobufacini, even at doses up to eight times higher than typically used in China, did not exhibit any dose-limiting toxicity, supporting the safety of CC for advanced CRC. Consequently, these findings suggest that CC combined with first-line treatment may improve clinical efficacy in stage III–IV colorectal cancer.

In traditional Chinese medicine theory, cinobufacini is believed to have the effects of clearing heat and detoxifying, reducing swelling, and resolving blood stasis (Qi et al., 2010). Recent pharmacological studies have shown that cinobufacini can promote tumor cell apoptosis, reverse multidrug resistance, and modulate the immune response (Zuo et al., 2024). A previous preclinical study reported that cinobufacini inhibited colon cancer invasion and metastasis by suppressing the Wnt/β-catenin signaling pathway and downregulating EMT-related genes, including MMP9, MMP2, N-cadherin, and Snail (Wang et al., 2020). Furthermore, Bufalin, the main active monomer in cinobufacini (Li M. et al., 2022; Wang et al., 2023) demonstrates anti-tumor properties through various mechanisms. These mechanisms involve inhibiting angiogenesis through the SRC-3/HIF-1α and STAT3 pathways (Yuan et al., 2022; Fang K. et al., 2021) as well as inhibiting the JAK-STAT3 pathway and activating mitochondrial ROS-mediated caspase-3 to induce tumor cell apoptosis (Zhu et al., 2012; Wu et al., 2019). Moreover, bufalin suppresses CRC cell proliferation and metastasis by inhibiting the c-Kit/Slug signaling axis and the STAT3 pathway (Wang et al., 2023; Ding et al., 2022). In addition, bufalin can regulate the CD133/nuclear factor-κB/MDR1 and SRC-3/MIF pathways to mitigate chemotherapy resistance in CRC (Zhan Y. et al., 2020; Chen et al., 2021). Besides, other active ingredients isolated from the dried skin of *Bufo gargarizans*, including resibufogenin, telocinobufagin, and cinobufagin, have also demonstrated notable antitumor activity (Liang et al., 2016; Jia et al., 2022; Ha et al., 2018; Li et al., 2019; Hu and Luo, 2024). In the meantime, cionbufagin can significantly increase the population of CD4^+^CD8^+^ double-positive T cells, promote the phagocytosis and proliferation of macrophages, and regulate the levels of immune cytokines including interleukin-2, interleukin-4, interleukin-10, and interferon-γ (Zhao et al., 2024; Wang et al., 2011; Yu et al., 2015). Therefore, the elucidation of these mechanisms supported our conclusions at the molecular level.

The study has several limitations that should be noted. Firstly, the RCTs included in this study had small sample sizes and were conducted only in China, which may limit the generalizability of the findings and increase the risk of potential bias, including small-study effects that may lead to inflated pooled effect estimates. In addition, given the predominance of small-sample trials, the possibility that pooled effect estimates may be inflated cannot be completely excluded. Secondly, all of the studies exhibited some deficiencies in randomization, allocation concealment, and blinding, resulting in a decrease in the overall quality of evidence. These methodological limitations may introduce performance, detection, and reporting bias, potentially leading to an overestimation of treatment effects, particularly for DCR and immune-related indicators. Moreover, the 12 RCTs included in this article did not report information on the primary site and molecular subtype of CRC. A previous study has shown that the median survival time for primary colorectal tumors located on the right side is approximately half that of those on the left (Venook et al., 2016), highlighting the significant impact of the primary location of CRC on treatment efficacy. Furthermore, various molecular subtypes including KRAS, NRAS, and BRAF genes, also play a crucial role in determining patient prognosis (Biller and Schrag, 2021). The absence of these crucial details hinders a comprehensive evaluation of cinobufacini’s efficacy in treating different molecular subtypes of advanced colorectal cancer. Furthermore, evidence regarding the long-term efficacy of CC is still limited. This is partly because most outcomes assessed in this meta-analysis, including disease control rate, objective response rate, tumor markers, and immune-related indicators, are surrogate or short-term endpoints, which do not allow definitive conclusions regarding overall survival or progression-free survival. Only one high-quality, randomized, phase II study in patients with stage II-III CRC reported a prolonged 3-year DFS after adjuvant cinobacillus treatment, suggesting a potential survival benefit.

Given the limited number and subpar quality of the randomized controlled trials (RCTs) analyzed, it is imperative to conduct more RCTs with rigorously designed, long-term follow-up, clear molecular subtype, and multi-center participation to confirm the positive effects and enhance the evidence strength of cinobufacini for advanced colorectal cancer.

## 5 Conclusion

Overall, this meta-analysis suggests that cinobufacini capsules, when used as an adjunct to oxaliplatin-based chemotherapy, may offer potential benefits in patients with advanced colorectal cancer, particularly in improving short-term treatment response and tolerability. These findings support the possible role of cinobufacini as a complementary therapeutic option. However, the current evidence is limited by the generally low methodological quality of the available randomized controlled trials, potential bias risks, and the reliance on surrogate or short-term endpoints. Future well-designed, multicenter randomized controlled trials with larger sample sizes, longer follow-up periods, and clearly defined molecular subtypes are needed to determine the long-term clinical value of cinobufacini, including its impact on overall survival and progression-free survival.

## Data Availability

The original contributions presented in the study are included in the article/supplementary material, further inquiries can be directed to the corresponding author.
